# Efficacy and factors of 3D-printed guide-assisted hollow screw internal fixation for femoral neck fractures in the elderly

**DOI:** 10.1080/07853890.2026.2634458

**Published:** 2026-03-25

**Authors:** Jiayue Bai, Pengchao Lin, Jinghang Cheng, Sisi Jing, Cui Wang, Jing Li, Kangning Hao, Weijie Yuan, Jiangyong Wang

**Affiliations:** a Trauma Department Three, Third Hospital of Shijiazhuang, Shijiazhuang, Hebei Province, China; b Joint Surgery Department, Gaocheng People’s Hospital, Shijiazhuang, Hebei Province, China; c TCM Pharmacy, Shijiazhuang Third Hospital, Shijiazhuang, Hebei Province, China; d Department of Nursing, Shijiazhuang Geriatric Hospital, Shijiazhuang, Hebei Province, China

**Keywords:** Elderly patients, femoral neck fractures, hip function, hollow screw internal fixation, 3D-printed guide templates

## Abstract

**Background:**

Femoral neck fractures in the elderly present significant challenges due to the complexity of the fractures and the fragility of patients. Traditional hollow screw internal fixation (HSIF) necessitates precise screw placement, which can be difficult due to compromised bone quality. This study explores the efficacy of 3D-printed percutaneous guide templates (3D-PPGTA) in assisting HSIF for improved surgical outcomes.

**Methods:**

A retrospective analysis was conducted on 233 elderly patients with femoral neck fractures treated between June 2015 and December 2023. Patients were divided into HSIF (*n* = 128) and 3D-PPGTA (*n* = 105) groups. Outcomes were assessed using the Oxford Hip Score (OHS) and Short-Form Health Survey (SF-36), evaluating hip function, quality of life, and surgical success.

**Results:**

One-year post-surgery, the 3D-PPGTA group showed significantly higher OHS (35.73 ± 1.65) than the HSIF group (27.41 ± 1.07, *p <* 0.001), indicating superior hip function. The SF-36 revealed significantly better scores in the 3D-PPGTA group for Physical Functioning (67.66 ± 7.36 vs. 65.26 ± 7.44, *p* = 0.015), Bodily Pain (66.11 ± 5.21 vs. 64.16 ± 5.88, *p* = 0.009), General Health (54.89 ± 5.23 vs. 52.88 ± 4.56, *p* = 0.002), and Vitality (51.12 ± 4.98 vs. 49.76 ± 4.11, *p* = 0.026). Factors such as shorter time to surgery, delayed weight-bearing, and quicker fracture healing were associated with better outcomes in subgroup analysis within the 3D-PPGTA cohort (*p <* 0.05). The 3D-PPGTA procedure was associated with improved functional outcomes without increasing surgical risks.

**Conclusion:**

The use of 3D-printed guide templates for assisted internal fixation is associated with improved functional recovery and quality of life in elderly patients with femoral neck fractures. The potential for enhanced operative precision warrants further investigation with radiographic metrics.

## 1. Introduction

Femoral neck fractures, a prevalent concern among the elderly population, pose significant challenges in orthopedic practice due to their complex nature and the inherent fragility of the patients affected [1,2]. These fractures, often resulting from low-energy mechanisms such as falls, were associated with high morbidity and mortality rates [3,4]. As the global population ages, the incidence of hip fractures was anticipated to rise, necessitating advancements in surgical interventions aimed at optimizing outcomes and enhancing the quality of life for patients.

Traditional methods such as hollow screw internal fixation (HSIF) have been the mainstay treatment for stabilizing femoral neck fractures, particularly in non-displaced or minimally displaced types [5,6]. While effective, these techniques require precise screw placement to ensure optimal biomechanical stability, an aspect that can be challenging given the intricate anatomy and compromised bone quality commonly seen in aged patients. Inaccuracies during screw placement can lead to inadequate fracture fixation, malunion, or non-union, ultimately impacting the patient’s recovery trajectory and functional outcomes [7,8].

The advent of 3D printing technology in the medical field has opened new avenues for enhancing surgical precision and personalization [9–11]. This innovative approach allows for the creation of patient-specific surgical aids, such as 3D-printed percutaneous guide templates (3D-PPGTA), which were designed based on preoperative imaging [12,13]. These templates serve as anatomical references during surgery, guiding the placement of orthopedic hardware with unprecedented accuracy. Utilizing 3D-printed guides for surgical interventions addresses the variability inherent in human anatomy, thus potentially improving the success rate of fracture fixations [14,15].

The current study aims to explore the efficacy and influencing factors of using 3D-PPGTA for assisting HSIF in elderly patients with femoral neck fractures. While existing literature, including studies on orthopedic applications and specific fracture fixations [16–18], substantiates the technical feasibility and potential of 3D printing, comprehensive analyses evaluating its impact on long-term functional outcomes and clinical recovery dynamics (e.g. time to healing, weight-bearing protocols) in a substantial cohort of elderly femoral neck fracture patients remain limited. Thus, this investigation seeks to contribute by providing empirical evidence focused on these patient-centered clinical outcomes and the factors influencing them within a 3D-guided surgical protocol.

## 2. Materials and methods

### 2.1. Inclusion and exclusion criteria

The study included patients who met the following criteria: they had a confirmed fracture of the femoral neck through anteroposterior and lateral hip radiographs, computed tomography (CT), or magnetic resonance imaging (MRI); they were aged 55 years or older; they had a follow-up period exceeding one year; and they possessed complete medical records and follow-up data.

Patients were excluded from the study if they had concurrent fractures or major injuries (e.g. significant head, thoracic, or abdominal trauma requiring immediate intervention) in other regions; were diagnosed with high-risk infectious diseases; exhibited cognitive impairments or psychiatric disorders; had cancer; or had undergone hip surgery within the last year.

### 2.2. Study design

A retrospective analysis was performed on 233 elderly patients with femoral neck fractures who were admitted to our hospital from June 2015 to December 2023. These patients were divided into two groups according to whether their fixation procedure was assisted by a 3D-PPGTA. The group that received HSIF without the guide template was termed the HSIF Group (*n* = 128). Conversely, the group that underwent HSIF with 3D-PPGTA assistance was termed the 3D-PPGTA Group (*n* = 105). The decision to use a 3D-PPGTA was primarily based on patient preference after detailed explanation of both techniques, coupled with the availability of the guide template at the time of surgery (as its preparation required additional time). All surgical procedures, in both groups, were performed or directly supervised by the same team of three senior attending trauma orthopedic surgeons, each with over 10 years of experience in hip fracture management. This team was proficient in both conventional and 3D-guided techniques. The distribution of cases among these surgeons was balanced between the two groups to mitigate the potential bias of operator skill influencing the outcomes.

To evaluate hip function, pain levels, and surgical outcomes, the Oxford Hip Score (OHS) was administered on the first day and one year following surgery. This tool comprises 12 items, each rated on a Likert scale ranging from 0 to 4; a score of 0 signifies the most severe problem, whereas a score of 4 indicates no problem. The total score, which was the sum of all item scores, ranges from 0 to 48; higher scores reflect better hip function. The Cronbach’s alpha for this questionnaire was recorded at 0.91 [19].

Within the 3D-PPGTA group, patients were further categorized based on their OHS at one-year post-surgery. Those with a score of 30 or higher were classified into the good outcome group (*n* = 51), while those with scores below 30 were assigned to the poor outcome group (*n* = 54) [20].

### 2.3. Treatment approach

#### 2.3.1. HSIF group

Patients in the HSIF group underwent conventional HSIF based on standard preoperative radiographs (anteroposterior and lateral views) and CT scan review in two-dimensional slices. Under general anesthesia, patients were placed in a supine position. Fracture reduction was achieved through manual manipulation combined with traction of the affected limb. Once satisfactory fracture reduction was confirmed using C-arm X-ray fluoroscopy, three Kirschner wires (K-wires) were inserted percutaneously in an inverted triangular formation along the femoral neck’s long axis. Following verification of the K-wires’ satisfactory positioning *via* fluoroscopy, holes were drilled, and depths measured before sequential insertion of three hollow screws. The traction frame was then relaxed to compress the screws. After confirming satisfactory fracture reduction and fixation through fluoroscopy, all surgical instruments and sponges were accounted for, and the wound was irrigated, sutured, and dressed. All cannulated screws used had a diameter of 6.5 mm. Their lengths were selected intraoperatively based on fluoroscopic measurements to achieve appropriate purchase in the femoral head without penetrating the articular surface.

#### 2.3.2. 3D-PPGTA group

**Figure 1.**
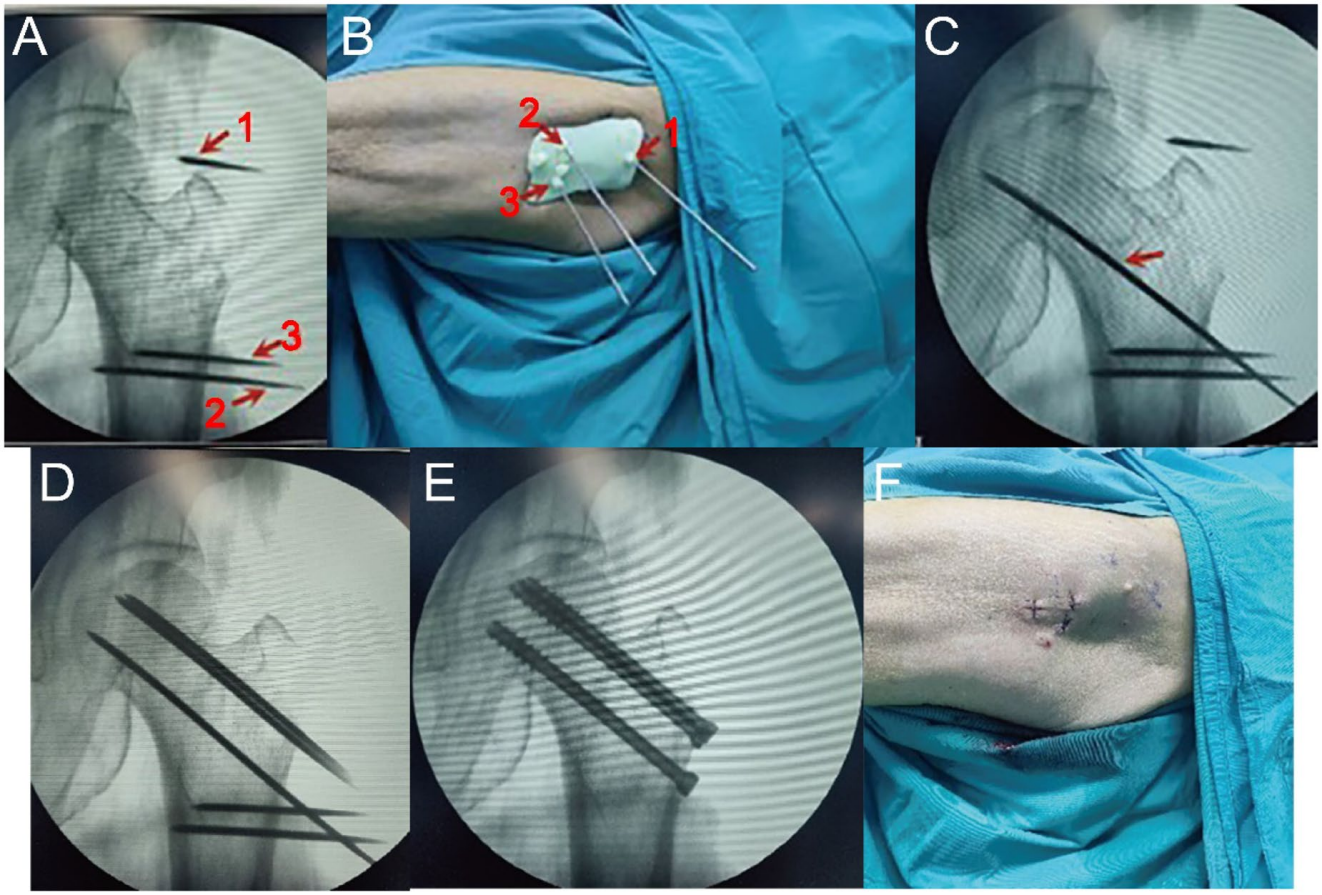
Schematic illustration of key surgical steps for hollow screw internal fixation assisted by the 3D-printed percutaneous guide template. (A) Insertion of three K-wires under fluoroscopic guidance (1: greater trochanter apex; 2: anterior femoral shaft; 3: posterior femoral shaft). (B) Fixation of the guide plate onto the skin using the K-wires. (C) Insertion of the calcar guidewire through the guide plate channel (arrow). (D) Sequential insertion of the remaining two guidewires. (E) Placement of three cannulated screws and removal of guidewires. (F) Final wound closure.

In the 3D-PPGTA group, patients received HSIF with the aid of a 3D-PPGTA. A schematic illustration depicting the key steps is provided in Figure 1. Preoperative CT scans of the fractures were performed with a 1 mm slice thickness, followed by 3D reconstruction. Medical computer-aided design software was used to create virtual internal fixation and reduction designs for 3D printing. The 3D reconstruction and virtual planning were interactively reviewed and approved by the attending surgeon prior to printing, allowing for a comprehensive three-dimensional understanding of fracture morphology, fragment positions, and optimal screw trajectory planning. A navigation template was designed and printed to guide the placement of hollow screws post-reduction. These data were input into a 3D printer (3D Systems, Shanghai Fofei Technology Development Co., Ltd., China) to produce a percutaneous guide template model and a 1:1 scale model of the affected femur. The guides were printed using a biocompatible, medical-grade photopolymer resin suitable for sterilization. After printing, each patient-specific guide was cleaned and then sterilized using a validated low-temperature sterilization process, specifically hydrogen peroxide plasma sterilization (STERRAD^®^ system), in accordance with our hospital’s protocol for heat-sensitive surgical instruments. A preoperative simulation of the fracture internal fixation was conducted on the 3D model to determine the optimal placement and insertion method for the hollow screws.

The infrastructure for creating the 3D guides involved a collaborative workflow. Based on the preoperative CT data, 3D reconstruction and virtual surgical planning, including fracture reduction and screw trajectory design, were performed using medical image processing and computer-aided design (CAD) software (E-3D Digital Medical Technology Co., Ltd., China). The patient-specific guide template was then fabricated using a stereolithography (SLA) 3D printer with a biocompatible, sterilizable resin material. Prior to surgery, the guide was sterilized according to the hospital’s standard protocol for heat-sensitive polymer instruments, using a validated low-temperature sterilization process (hydrogen peroxide plasma or ethylene oxide). The entire process, from CT acquisition to a sterilized guide ready for surgery, typically required approximately 24–48 h, allowing for integration into the surgical schedule for planned procedures.

Under satisfactory anesthesia, the patient was placed supine on an orthopedic surgical traction bed with the affected limb extended and the patella facing upwards. Referring to surface landmarks on the skin, two 2.5 mm K-wires were percutaneously inserted horizontally from the apex of the greater trochanter parallel to the femoral shaft axis (without penetrating the bone). Similarly, two additional K-wires of the same specifications were percutaneously inserted horizontally from the lateral side of the femur at the distal level of the lesser trochanter, closely adjacent to the femoral cortex, reaching anterior and posterior positions relative to the lesser trochanter (without penetrating the bone). These four K-wires, anchored in the soft tissues at the planned entry points, were used to initially position and secure the 3D-printed guide plate onto the patient’s skin. The 3D-printed guide plate, pre-drilled for positioning, was then fixed onto the femoral shaft by threading it over the tails of the four K-wires. A skin incision was made around the K-wires. Fluoroscopic examination using a C-arm X-ray machine was performed to confirm the accuracy of the guide plate’s position, particularly ensuring alignment with the predetermined screw paths. Minor adjustments were made by repositioning the K-wires within the soft tissue to achieve optimal alignment, ensuring the guide plate was stably positioned with its inner surface closely opposed to the contour of the femoral bone surface prior to guidewire insertion.

Under traction, the fracture was manually reduced, and fluoroscopic confirmation was obtained to ensure proper alignment and stability of the fracture ends. Once satisfactory reduction was achieved, the guidewire for the distal femoral neck position was inserted first, followed by sequentially inserting the remaining guidewires through the openings in the guide plate, with each insertion confirmed by fluoroscopy to ensure that the guidewires followed the predetermined screw paths into the femur. Any deviations in guidewire position were corrected promptly by repositioning and reinsertion until the desired position was achieved. After confirming the correct position of the guidewires, all K-wires and the guide plate were removed. Drilling and tapping were performed along the guidewires, the traction frame was loosened, and cannulated screws were inserted along the guidewires, ensuring correct placement and appropriate length before screw insertion to avoid compromising fixation efficacy. After all screws were inserted, the guidewires were removed, and final fluoroscopic confirmation was performed to verify the reduction and screw fixation outcomes. The wound was irrigated, sutured, and dressed. Similarly, 6.5 mm diameter cannulated screws were used. Their lengths were preoperatively planned using the 3D software and confirmed intraoperatively, aiming for optimal subchondral positioning as per the virtual plan.

In the present study, all 105 patient-specific 3D-printed guides were successfully utilized during the planned surgical procedures. There were no instances where a guide had to be abandoned intraoperatively due to improper fit, breakage, or failure to achieve satisfactory alignment. The preoperative simulation on the 3D-printed bone model and the intraoperative fluoroscopic confirmation of guide plate positioning prior to guidewire insertion were crucial steps that ensured the guide’s usability and accuracy in every case.

### 2.4. Data collection

Patient data were gathered from the medical record system, encompassing demographic information, OHS, Short-Form Health Survey (SF-36) score, intraoperative parameters, and postoperative status and complications.

The Garden classification system distinguishes femoral neck fractures into four types [21]: Type I describes incomplete, stable fractures with valgus impaction; Type II involves complete yet non-displaced fractures with aligned trabecular groups; Type III consists of partially displaced fractures with varus deformity affecting all three trabecular groups; and Type IV pertains to completely displaced fractures with no contact between fracture fragments.

Postoperative weight-bearing time was defined as the period from the end of surgery to when patients were allowed to partially or fully bear weight on the affected limb for activities like standing or walking. This timing was essential to ensure proper healing while reducing the risk of complications.

### 2.5. Assessments of quality of life

To assess the impact of the intervention on patients’ quality of life, the SF-36 was employed. This questionnaire covers eight core domains: Physical Functioning (PF), Role Limitations due to Physical Health Problems (RP), Bodily Pain (BP), General Health (GH), Vitality (VT), Social Functioning (SF), Role Limitations due to Emotional Problems (RE), and Mental Health (MH). Scores for each domain range from 0 to 100, with higher scores signifying a better quality of life. The SF-36 exhibits strong internal consistency reliability, with Cronbach’s alpha coefficients ranging from 0.72 to 0.88 [22].

### 2.6. Ethics statement

The study received approval from the Institutional Review Board and Ethics Committee of Shijiazhuang Third Hospital (Approval No.2023-003), and abided by the ethical guidelines of the Declaration of Helsinki. Since only de-identified patient data were used, posing no potential harm or impact on the patients, the requirement for informed consent was waived. This waiver was granted by the Institutional Review Board and Ethics Committee in accordance with the regulatory and ethical standards applicable to retrospective research studies.

### 2.7. Statistical analysis

Data were analyzed using SPSS 29.0 statistical software (SPSS Inc., Chicago, IL, USA). Categorical data were presented as frequencies and percentages [*n* (%)] and analyzed using chi-square tests. The standard chi-square test was applied when the sample size was ≥40 and the theoretical frequency (*T*) was ≥5. In cases where the sample size was ≥40 but 1≤*T* < 5, a corrected chi-square test was employed. For sample sizes < 40 or where *T* < 1, Fisher’s exact test was utilized. Continuous variables were assessed for normality using the Shapiro–Wilk test. Normally distributed continuous data were expressed as mean and standard deviation (*X* ± *s*). A *P* value of < 0.05 was considered statistically significant. To assess correlations involving a mix of continuous and ordinal/categorical variables with the dichotomized outcome, Spearman’s rank correlation coefficient (ρ) was used. The efficacy of the 3D-PPGTA HSIF was examined through correlation analysis, univariate analysis, and multivariate analysis. For the univariate and multivariate analyses examining factors associated with efficacy within the 3D-PPGTA group, binary logistic regression was employed. Variables yielding a *P* value <0.1 in the univariate analysis were considered candidates for inclusion in the multivariate logistic regression model. The final multivariate model was constructed using a forward stepwise (likelihood ratio) method to identify independent factors.

## 3. Results

### 3.1. Basic Data and curative effect of HSIF group and 3D-PPGTA group

The mean age was similar between groups, with no statistically significant difference (HSIF: 62.65 ± 3.02 years, 3D-PPGTA: 63.13 ± 2.94 years; *p =* 0.223) (Table 1). Similarly, body mass index (BMI) did not differ significantly between the HSIF group (22.84 ± 2.14 kg/m^2^) and the 3D-PPGTA group (22.39 ± 1.84 kg/m^2^; *p =* 0.089). Gender distribution was comparable, with males comprising 50.78% of the HSIF group and 53.33% of the 3D-PPGTA group (*p =* 0.698). The prevalence of smoking history, drinking history, and comorbidities such as hypertension and diabetes were not significantly different between groups (*p* > 0.05). Marital status, monthly income levels, Garden fracture classification, and cause of fracture also showed no significant differences (*p* > 0.05). Time from admission to surgery was nearly identical between the groups, with the HSIF group averaging 1.54 ± 0.35 days and the 3D-PPGTA group 1.58 ± 0.39 days (*p =* 0.436). Overall, demographic variables were well-matched between the two treatment cohorts, with no statistically significant differences observed.

**Table 1.** Comparison of demographic characteristics between two groups.

Parameters	HSIF group (*n* = 128)	3D-PPGTA group (*n* = 105)	*t*/χ^2^	*P*
Age (years)	62.65 ± 3.02	63.13 ± 2.94	1.223	0.223
BMI (kg/m²)	22.84 ± 2.14	22.39 ± 1.84	1.707	0.089
Male/female [*n* (%)]	65 (50.78%)/63 (49.22%)	56 (53.33%)/49 (46.67%)	0.151	0.698
Smoking history [*n* (%)]	32 (25%)	27 (25.71%)	0.016	0.901
Drinking history [*n* (%)]	26 (20.31%)	20 (19.05%)	0.058	0.809
Hypertension [*n* (%)]	30 (23.44%)	23 (21.9%)	0.077	0.781
Diabetes [*n* (%)]	18 (14.06%)	12 (11.43%)	0.357	0.550
Marital status (married/unmarried or divorced) [*n* (%)]	113 (88.28%)/15 (11.72%)	96 (91.43%)/9 (8.57%)	0.618	0.432
Monthly average income (<3000/3000–6000/>6000) [*n* (%)]	25 (19.53%)/67 (52.34%)/36 (28.12%)	20 (19.05%)/45 (42.86%)/40 (38.1%)	2.845	0.241
Garden type (I, II/III, IV) [*n* (%)]	87 (67.97%)/41 (32.03%)	71 (67.62%)/34 (32.38%)	0.003	0.955
Fracture cause (motor vehicle accident/fall injury/crush injury) [*n* (%)]	102 (79.69%)/11 (8.59%)/15 (11.72%)	80 (76.19%)/7 (6.67%)/18 (17.14%)	1.566	0.457
Time from admission to surgery (days) [*n* (%)]	1.54 ± 0.35	1.58 ± 0.39	0.780	0.436

BMI: body mass index.

At 1-day post-surgery, the mean OHS was similar between the groups, with the HSIF group scoring 19.24 ± 1.43 and the 3D-PPGTA group scoring 19.35 ± 1.71, showing no statistical significance (*p =* 0.593) (Table 2). However, at 1-year post-surgery, the 3D-PPGTA group demonstrated a significantly higher OHS (35.73 ± 1.65) compared to the HSIF group (27.41 ± 1.07), with a *P* value of less than 0.001, indicating a substantial improvement in hip function associated with the 3D-PPGTA method over time.

**Table 2.** Comparison of OHS between two groups.

Parameters	HSIF group (*n* = 128)	3D-PPGTA group (*n* = 105)	*T*	*P*
1-day post-surgery	19.24 ± 1.43	19.35 ± 1.71	0.536	0.593
1-year post-surgery	27.41 ± 1.07	35.73 ± 1.65	44.485	<0.001

OHS: Oxford Hip Score.

**Figure 2.**
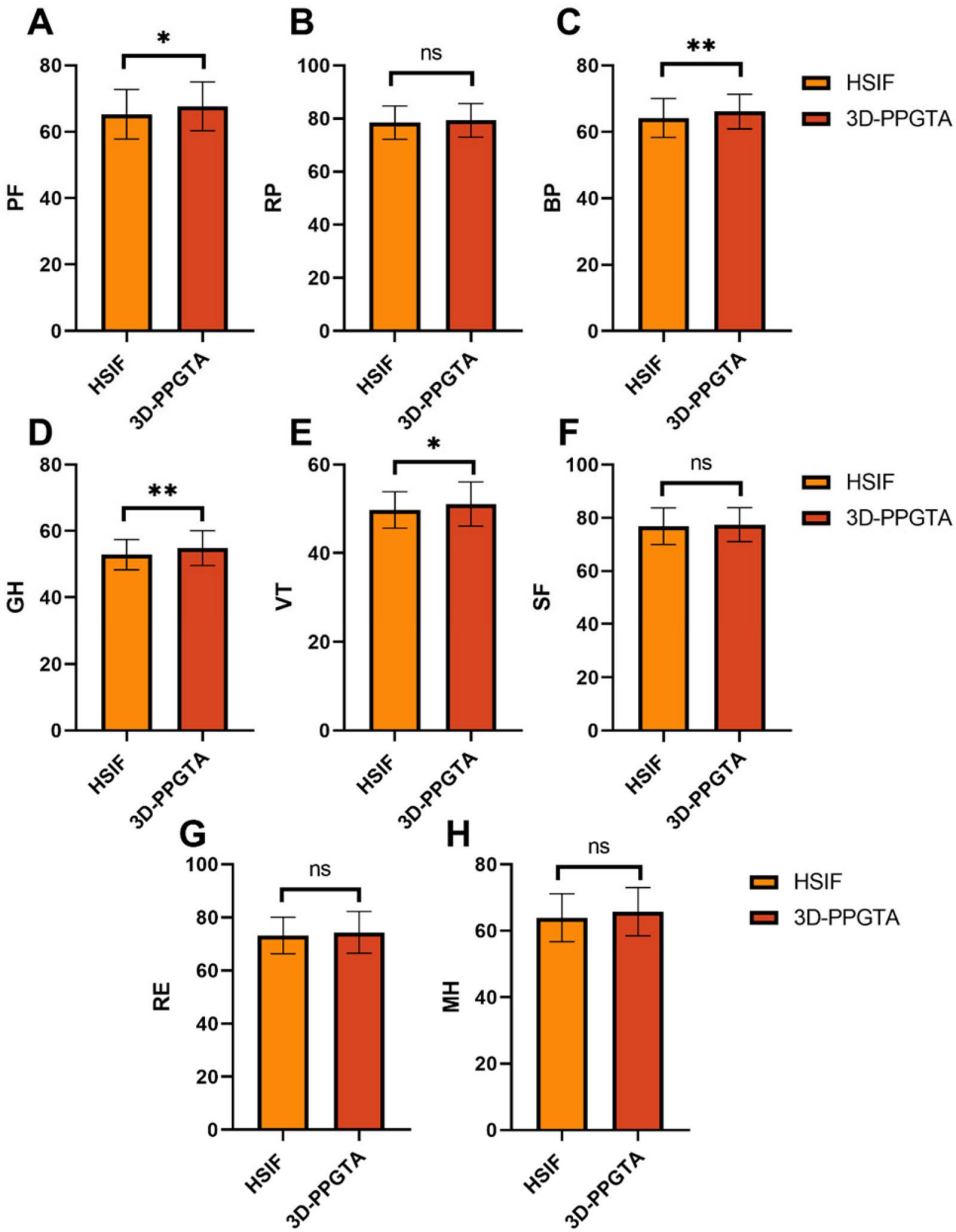
Comparison of SF-36 score 1-year post-surgery between two groups. Data are presented as bar charts showing the mean ± standard deviation. (A) PF (B) RP (C) BP (D) GH (E) VT (F) SF (G) RE (H) MH ns: no significant difference; *p < 0.05; **p < 0.01. SF-36: Short-Form Health Survey; PF: Physical Functioning; RP: Role-Physical; BP: Bodily Pain; GH: General Health; VT: Vitality; SF: Social Functioning; RE: Role-Emotional; MH: Mental Health.

There were statistically significant improvements in the 3D-PPGTA group compared to the HSIF group in PF (PF: 67.66 ± 7.36 vs 65.26 ± 7.44, *p =* 0.015) (Figure 2A), BP (BP: 66.11 ± 5.21 vs 64.16 ± 5.88, *p =* 0.009) (Figure 2C), GH (GH: 54.89 ± 5.23 vs 52.88 ± 4.56, *p =* 0.002) (Figure 2D), and VT (VT: 51.12 ± 4.98 vs 49.76 ± 4.11, *p =* 0.026) (Figure 2E). However, no significant differences were observed between the groups in role-physical (RP: *p =* 0.289) (Figure 2B), SF (SF: *p =* 0.508) (Figure 2F), role-emotional (RE: *p =* 0.212) (Figure 2G), and MH (MH: *p =* 0.054) (Figure 2H), indicating comparable outcomes in these domains. Overall, the 3D-PPGTA group exhibited superior outcomes in several physical health domains, suggesting better overall recovery in these areas.

### 3.2. Baseline characteristics of the good and poor outcome subgroups within the 3D-PPGTA cohort

Patients within the 3D-PPGTA group (*n* = 105) were further categorized based on their OHS at one-year post-surgery. There were no statistically significant differences between the groups in age (good outcome: 62.76 ± 2.14 years vs poor outcome: 63.41 ± 2.25 years, *p =* 0.131), BMI, gender distribution, smoking and drinking history, prevalence of hypertension and diabetes, marital status, monthly average income, and the cause of fracture (*p* > 0.05) (Table 3). However, significant differences were noted in the Garden classification and the time from admission to surgery. Patients in the good outcome group were more likely to have a Garden type I or II classification (78.43% vs 57.41%, *p =* 0.021), and they had a shorter average time from admission to surgery (1.45 ± 0.24 days vs 1.61 ± 0.27 days, *p =* 0.002), suggesting these factors may influence surgical outcomes.

**Table 3.** Comparison of demographic characteristics between two groups.

Parameters	Good outcome group (*n* = 51)	Poor outcome group (*n* = 54)	*t*/χ^2^	*P*
Age (years)	62.76 ± 2.14	63.41 ± 2.25	1.522	0.131
BMI (kg/m²)	22.04 ± 1.53	22.45 ± 1.48	1.413	0.161
Female/male [*n* (%)]	27 (52.94%)/24 (47.06%)	29 (53.7%)/25 (46.3%)	0.006	0.938
Smoking history [*n* (%)]	12 (23.53%)	15 (27.78%)	0.248	0.619
Drinking history [*n* (%)]	10 (19.61%)	10 (18.52%)	0.020	0.887
Hypertension [*n* (%)]	12 (23.53%)	11 (20.37%)	0.153	0.696
Diabetes [*n* (%)]	5 (9.8%)	7 (12.96%)	0.259	0.611
Marital status (married/unmarried or divorced) [*n* (%)]	48 (94.12%)/3 (5.88%)	48 (88.89%)/6 (11.11%)	0.369	0.543
Monthly average income (<3000/3000–6000/>6000) [*n* (%)]	10 (19.61%)/22 (43.14%)/19 (37.25%)	10 (18.52%)/23 (42.59%)/21 (38.89%)	0.037	0.982
Garden type (I, II/III, IV) [*n* (%)]	40 (78.43%)/11 (21.57%)	31 (57.41%)/23 (42.59%)	5.295	0.021
Fracture cause (motor vehicle accident/fall injury/crush injury) [*n* (%)]	37 (72.55%)/3 (5.88%)/11 (21.57%)	43 (79.63%)/4 (7.41%)/7 (12.96%)	1.397	0.497
Time from admission to surgery (days)	1.45 ± 0.24	1.61 ± 0.27	3.219	0.002

### 3.3. Intraoperative parameters

**Figure 3.**
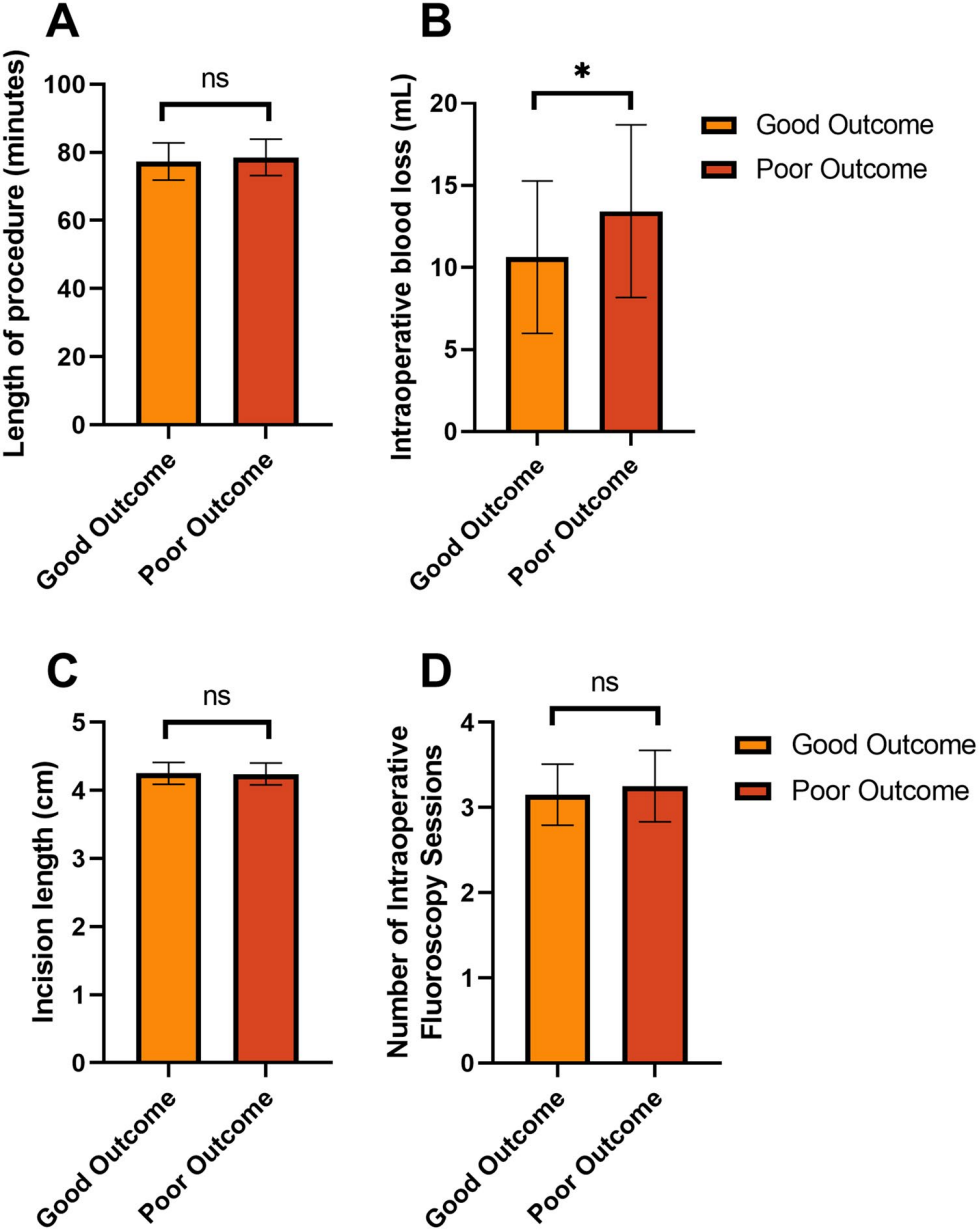
Comparison of intraoperative parameters between good and poor outcome subgroups in the 3D-PPGTA cohort. Data are presented as bar charts showing the mean ± standard deviation. (A) Length of procedure, (B) intraoperative blood loss, (C) incision length, (D) number of intraoperative fluoroscopy sessions ns: no significant difference: *: *p <* 0.05.

The good outcome group had significantly lower blood loss (10.63 ± 4.64 mL) compared to the poor outcome group (13.43 ± 5.26 mL) (*p =* 0.005) (Figure 3B). However, no significant differences were found in the length of the procedure (*p =* 0.264) (Figure 3A), incision length (*p =* 0.702) (Figure 3C), and the number of intraoperative fluoroscopy sessions (*p =* 0.203) (Figure 3D), as these parameters were similar between the two groups. This suggests that while most intraoperative metrics were comparable, reduced blood loss was associated with better outcomes.

### 3.4. Postoperative status

A statistically significant difference was found in the timing of postoperative weight-bearing: the good outcome group had a higher percentage of patients who began weight-bearing at or beyond 3 months (58.82%) compared to the poor outcome group (35.19%) (*p =* 0.015) (Table 4). Additionally, the time to complete fracture healing was significantly shorter in the good outcome group (7.02 ± 1.25 months) than in the poor outcome group (7.74 ± 1.22 months, *p =* 0.004). No significant difference was observed in the follow-up duration between the groups (*p =* 0.584), indicating similar long-term monitoring across the cohorts. These findings suggest that both delayed weight-bearing beyond 3 months and a shorter time to fracture healing were associated with better surgical outcomes.

**Table 4.** Comparison of postoperative status between two groups.

Parameters	Good outcome group (*n* = 51)	Poor outcome group (*n* = 54)	*t*/χ^2^	*P*
Postoperative weight-bearing time (<3 months/≥3 months) [*n* (%)]	21 (41.18%)/30 (58.82%)	35 (64.81%)/19 (35.19%)	5.888	0.015
Follow-up duration (months)	14.62 ± 1.45	14.78 ± 1.62	0.550	0.584
Time to complete fracture healing (months)	7.02 ± 1.25	7.74 ± 1.22	2.968	0.004

### 3.5. Complications

Across both groups, there were no statistically significant differences in the rates of incision infections (1.96% in the good outcome group vs 7.41% in the poor outcome group, *p =* 0.395), non-union of the bone (3.92% vs 11.11%, *p =* 0.308), pin-track infections (0% vs 3.7%, *p =* 0.501), sacral nerve injuries (0% vs 5.56%, *p =* 0.262), venous thrombosis (0% vs 5.56%, *p =* 0.262), and avascular necrosis of the femoral head (1.96% vs 5.56%, *p =* 0.651) (Table 5). These findings suggest that while complications occurred at varying frequencies, none were significantly associated with poorer outcomes in this cohort.

**Table 5.** Comparison of complications between two groups.

Parameters	Good outcome group (*n* = 51)	Poor outcome group (*n* = 54)	χ^2^	*P*
Incision infection [*n* (%)]	1 (1.96%)	4 (7.41%)	0.725	0.395
Does not heal of the bone [*n* (%)]	2 (3.92%)	6 (11.11%)	1.040	0.308
Pin-track infection [*n* (%)]	0 (0%)	2 (3.7%)	0.453	0.501
Sacral nerve injury [*n* (%)]	0 (0%)	3 (5.56%)	1.258	0.262
Venous thrombosis [*n* (%)]	0 (0%)	3 (5.56%)	1.258	0.262
Avascular necrosis of the femoral head [*n* (%)]	1 (1.96%)	3 (5.56%)	0.204	0.651

### 3.6. Correlation analysis of factors associated with outcome in the 3D-PPGTA group

Spearman’s correlation analysis was performed to examine the association between various parameters and the dichotomized outcome (good vs. poor) within the 3D-PPGTA group (Table 6). This analysis included only the patients from the 3D-PPGTA group (*n* = 105). Spearman’s correlation coefficients (ρ) were calculated to assess the strength and direction of the association between each potential influencing factor and the dichotomized surgical outcome (where Good Outcome = 0, Poor Outcome = 1). There was a negative correlation between Garden type classifications (I, II) and poor outcome (rho = –0.201, *p =* 0.040), indicating that less severe fracture types were associated with better outcomes. A positive correlation was observed between a longer time from admission to surgery and an increased likelihood of a poor outcome (rho = 0.305, *p =* 0.002), suggesting that shorter times to surgery may enhance recovery. Intraoperative blood loss was also positively correlated with poor outcome (rho = 0.222, *p =* 0.023), as was delayed postoperative weight-bearing beyond 3 months (rho = 0.237, *p =* 0.015), and a shorter time to complete fracture healing (rho = 0.254, *p =* 0.009).

**Table 6.** Correlation analysis of factors with poor outcome within the 3D-PPGTA group.

Parameters	Rho	*P*
Garden type (I, II/III, IV) [*n* (%)]	−0.201	0.040
Time from admission to surgery (days)	0.305	0.002
Intraoperative blood loss (mL)	0.222	0.023
Postoperative weight-bearing time (<3 months/≥3 months) [*n* (%)]	0.237	0.015
Time to complete fracture healing (months)	0.254	0.009

### 3.7. Univariate analysis of factors associated with outcome in the 3D-PPGTA group

Univariate binary logistic regression was performed with poor outcome as the dependent variable (poor outcome = 1, good outcome = 0) (Table 7). An odds ratio (OR) > 1 indicates increased odds of a poor outcome. Garden type (I, II) was inversely related to poor outcome, with a coefficient of −0.853 (*p =* 0.042) and an OR of 0.426, indicating that less severe fractures were associated with better results. The time from admission to surgery was a strong predictor, with a coefficient of 2.499 (*p =* 0.003) and an OR of 12.171, suggesting that longer time to surgery is associated with poor outcome. Intraoperative blood loss was also significant, with a coefficient of 0.015 (*p =* 0.030) and an OR of 1.016, indicating that greater blood loss is associated with slightly higher odds of a poor outcome. Postoperative weight-bearing time less than 3 months was harmful, with a coefficient of 0.968 (*p =* 0.016) and an OR of 2.632. Finally, a longer time to complete fracture healing worsened outcomes, with a coefficient of 0.474 (*p =* 0.005) and an OR of 1.606, highlighting the importance of timely recovery to successful surgical outcomes.

**Table 7.** Univariate logistic regression analysis of factors associated with poor outcome within the 3D-PPGTA group.

Parameters	Coefficient	Std error	Wald	*P*	OR	95%CI
Garden type (I, II/III, IV) [*n* (%)]	−0.853	0.419	2.036	0.042	0.426	0.184–0.959
Time from admission to surgery (days)	2.499	0.839	2.979	0.003	12.171	2.512–68.998
Intraoperative blood loss (mL)	0.015	0.007	2.168	0.030	1.016	1.002–1.031
Postoperative weight-bearing time (<3 months/≥3 months) [*n* (%)]	0.968	0.403	2.403	0.016	2.632	1.207–5.882
Time to complete fracture healing (months)	0.474	0.171	2.777	0.005	1.606	1.164–2.284

We included time to complete fracture healing in the regression analysis acknowledging its close relationship with functional outcome. While not a traditional preoperative predictor, it represents a key intermediate postoperative milestone. Its strong association with the one-year OHS in our analysis underscores that delayed healing is an important marker and potentially a contributing factor to inferior long-term functional results, highlighting the importance of optimizing conditions for timely union.

### 3.8. Multivariate analysis of factors associated with outcome in the 3D-PPGTA group

Variables with a *P* value < 0.1 in the univariate analysis were entered into the initial multivariate logistic regression model. The multivariate analysis of factors associated with Poor Outcome within the 3D-PPGTA group identified several independent risk factors (Table 8). Time from admission to surgery emerged as a critical independent risk factor for a poor outcome, with a coefficient of 2.540 (*p =* 0.008) and an odds ratio (OR) of 12.677. This indicates that a longer delay before surgery was strongly associated with increased odds of an inferior functional result, emphasizing the importance of expedited surgical management. Postoperative weight-bearing time of three months or longer was also associated with poor outcome, with a coefficient of 0.983 (*p =* 0.032) and an OR of 2.672. Similarly, a longer time to complete fracture healing was associated with worse results, reflected by a coefficient of 0.431 (*p =* 0.022) and an OR of 1.539. While intraoperative blood loss showed a slight trend toward significance (*p =* 0.061), its effect was not statistically confirmed. Garden type did not reach statistical significance in this analysis (*p =* 0.129), suggesting less impact when other factors were considered. These insights, derived from the 3D-PPGTA cohort, highlight factors that may be particularly important for optimizing outcomes when employing this precise, guide-assisted surgical technique for femoral neck fractures in the elderly.

**Table 8.** Multivariate analysis of factors associated with poor outcome within the 3D-PPGTA group.

Parameters	Coefficient	Std error	Wald Stat	*P*	OR	OR CI lower	OR CI upper
Garden type (I, II/III, IV) [*n* (%)]	−0.719	0.474	−1.517	0.129	0.487	0.192	1.234
Time from admission to surgery (days)	2.540	0.956	2.656	0.008	12.677	1.946	82.577
Intraoperative blood loss (mL)	0.015	0.008	1.874	0.061	1.015	0.999	1.032
Postoperative weight-bearing time (<3 months/≥3 months) [*n* (%)]	0.983	0.460	2.139	0.032	2.672	1.086	6.577
Time to complete fracture healing (months)	0.431	0.188	2.292	0.022	1.539	1.064	2.226

## 4. Discussion

The study investigates the efficacy of 3D-PPGTA HSIF (3D-PPGTA) compared to traditional HSIF methods in elderly patients with femoral neck fractures. The central finding of this study is the significantly better hip function and quality of life observed one year postoperatively in patients treated with 3D-PPGTA compared to conventional HSIF. OHS was significantly higher at one-year post-surgery in the 3D-PPGTA group compared to the HSIF group. This suggests that the use of 3D-printed templates facilitates more precise surgical interventions, which may lead to superior anatomical alignment and stabilization of the fracture. The superior functional outcomes observed suggest that the 3D template assistance may facilitate more accurate screw placement, which is a key factor hypothesized to optimize the biomechanical environment for fracture healing [23,24]. This precision was particularly vital in elderly patients, where bone quality may be compromised due to osteoporosis, making accurate fixation crucial for preventing complications such as malunion or non-union [25,26].

Another notable observation was the enhancement of several domains within the SF-36 seen in patients undergoing the 3D-PPGTA procedure. Improvements in PF, BP, GH, and VT suggest that patients experience not only better physical recovery but also an enhanced perceived quality of life. The mechanical stability afforded by precise screw positioning could contribute to this improvement by allowing earlier and more confident mobilization, thus preventing the cascade of health declines often associated with prolonged immobility in older adults [27]. Early and safe mobilization was essential in this demographic to maintain muscle strength, prevent thromboembolic events, and reduce the risk of pressure ulcers. Our finding of significantly better functional outcomes with 3D-PPGTA is consistent with the results reported by Gao et al. [17], who also found superior hip function scores in middle-aged and elderly patients using a similar 3D-printed navigation template.

The study’s correlation analysis highlights several influencing factors that modulate the efficacy of the 3D-PPGTA technique, particularly focusing on preoperative and postoperative timelines. The time from admission to surgery emerged as a critical modulator of outcomes. A shorter time to surgery for patients receiving 3D-PPGTA significantly improved surgical efficacy, underscoring the importance of timely intervention in hip fractures, which were time-sensitive emergencies. Minimizing the preoperative waiting period may help mitigate the risk of further displacement and soft-tissue compromise, factors of particular concern in elderly patients with slower reparative processes. Although the absolute difference in waiting time between outcome subgroups was on the order of hours (mean difference ∼0.16 days), its strong association with outcome in the multivariate model suggests that even relatively modest delays may be clinically meaningful in this vulnerable population, reinforcing the principle of expeditious surgery for hip fractures.

Postoperative weight-bearing strategies also significantly influenced outcomes. Patients who engaged in delayed weight-bearing beyond three months demonstrated better recovery. In our protocol, weight-bearing encompassed the initiation of either partial or full loading as tolerated. The observed association between delaying this initiation beyond three months and better outcomes suggests that, in the context of this precise 3D-guided fixation, a more conservative rehabilitation approach—allowing for longer protected consolidation—might be beneficial. This may reflect the technique’s ability to achieve such stable fixation that premature loading, rather than instability, becomes a potential risk factor for micromotion [28].

Interestingly, Garden classification appeared less significant in multivariate analyses, suggesting that fracture displacement severity might become a less dominant factor when precision fixation technologies were utilized. This may indicate that 3D-PPGTA was particularly adept at managing complex fractures where displacement was significant, neutralizing its potential adverse influence on healing outcomes. This represents a significant shift in hip fracture management philosophy, where traditionally, less severe fractures tend to correlate with better recovery outcomes [29,30].

The intraoperative metrics further elucidate the potential advantages conferred by the use of 3D guides. Although intraoperative blood loss was not statistically significant in the multivariate analysis, the trend suggests that reduced visibility and structural disruption might result from the precision the guides provide. A potential benefit was shorter surgical times and less tissue manipulation, reducing the overall physiological stress and enhancing recovery trajectories.

Regarding surgical complications, the lack of significant differences in incidences such as infections or avascular necrosis highlights that the guide-assisted approach does not inadvertently increase surgical risks, affirming its safety profile. The precise guidance provided by the templates may lead to reduced risk of soft-tissue and neurovascular injuries, often a concern in minimally invasive procedures [31].

The analytical approach of this study was two-fold. First, we compared the primary clinical outcomes (OHS, SF-36) between the 3D-PPGTA and HSIF groups to evaluate the overall efficacy of the new technology. Second, recognizing that the 3D-PPGTA represents a novel and technically distinct method, we conducted an in-depth exploration specifically within this cohort. This secondary analysis aimed to identify which patient characteristics, surgical nuances, or postoperative rehabilitation factors were associated with optimal outcomes when using this precise, guide-assisted technique. This focused investigation is crucial for understanding how to best implement and optimize the 3D-PPGTA procedure in clinical practice.

These findings hold significant implications for the future of orthopedic surgery in elderly populations. The integration of 3D-printed technology into surgical protocols for femoral neck fractures addresses key challenges: precision, personalization, and time-efficient surgical care delivery [32–34]. Technological advancements facilitate personalized medicine—offering tailored surgical approaches considering patient-specific anatomy—and provide substantial educational tools for surgical teams. Surgeons can engage in preoperative planning and simulation, which may help in reducing the learning curve associated with technical procedures such as percutaneous screw fixation.

However, the study also presents limitations. This was a retrospective, non-randomized study. The assignment to the 3D-PPGTA or HSIF group was influenced by patient preference after detailed consultation and the real-time availability of the customized guide, rather than random allocation. Although our statistical analysis showed well-balanced baseline characteristics between the two groups, and all surgeries were performed by the same experienced team of surgeons to mitigate operator skill bias, unmeasured confounding factors may still exist. The potential for selection bias cannot be entirely ruled out. Therefore, the findings regarding the superiority of the 3D-PPGTA technique should be interpreted with caution. Future prospective randomized trials were necessary to validate these findings and explore long-term outcomes related to functional recovery and overall survival. We did not systematically assess or adjust for bone mineral density or the presence of osteoporosis in our cohort. Given that osteoporosis is prevalent in this age group and can influence screw purchase and fracture healing, this represents a potential confounding factor that future studies should address. While the 3D planning software allowed for virtual optimization of screw trajectory and spacing, postoperative radiographic measurements of inter-screw distance or precise distance to the subchondral surface were not performed in this clinical outcomes-focused study. These quantitative radiographic accuracy metrics represent an important area for future comparative research. The identified factors influencing outcome (surgical delay, weight-bearing timing) and the proposed mechanisms for the benefits of 3D guidance are derived from and specifically applicable to the 3D-PPGTA cohort. Their relevance to, or interaction with, outcomes following conventional HSIF cannot be determined from this study design. Furthermore, cost-analysis studies would be valuable in understanding the economic impact of incorporating 3D-printed guides into routine practice, looking at not just upfront costs but also longer-term savings through potentially reduced complication rates and faster patient mobility.

## 5. Conclusion

In conclusion, this study demonstrates that the use of 3D-printed guide templates for assisted internal fixation is associated with significantly improved functional recovery and quality of life in elderly patients with femoral neck fractures, as evidenced by superior Oxford Hip Scores and SF-36 outcomes compared to conventional fixation. Furthermore, exploratory analysis within the 3D-PPGTA cohort suggested that timely surgical intervention and a tailored postoperative rehabilitation protocol might be important for optimizing outcomes when employing this specific technology. These insights warrant further investigation in prospective studies. These insights pave the way for further exploration of how modern technology can be harnessed to improve clinical outcomes in orthopedic surgery, especially within vulnerable populations. As healthcare continues to evolve towards personalized care models, the interdisciplinary application of advanced technologies like 3D-PPGTA serves as a beacon for innovative and effective patient-centered solutions.

## Data Availability

The data that support the findings of this study are available from the corresponding author upon reasonable request.
